# Carnosine Supplementation Enhances Post Ischemic Hind Limb Revascularization

**DOI:** 10.3389/fphys.2019.00751

**Published:** 2019-07-02

**Authors:** Adjoa A. Boakye, Deqing Zhang, Luping Guo, Yuting Zheng, David Hoetker, Jingjing Zhao, Dheeraj Kumar Posa, Chin K. Ng, Huaiyu Zheng, Amit Kumar, Vijay Kumar, Michael F. Wempe, Aruni Bhatnagar, Daniel J. Conklin, Shahid P. Baba

**Affiliations:** ^1^Diabetes and Obesity Center, University of Louisville, Louisville, KY, United States; ^2^Department of Medicine, Envirome Institute, University of Louisville, Louisville, KY, United States; ^3^Department of Radiology, University of Louisville, Louisville, KY, United States; ^4^Department of Pharmaceutical Sciences, University of Colorado, Denver, Denver, CO, United States

**Keywords:** angiogenesis, Flk-1^+^/Sca-1^+^ cells, hind limb ischemia, HIF-1α, iron chelation, 4-hydroxy-nonenal, prolyl hydroxylases, peripheral arterial disease

## Abstract

High (millimolar) concentrations of the histidine containing dipeptide – carnosine (β-alanine-L-histidine) are present in the skeletal muscle. The dipeptide has been shown to buffer intracellular pH, chelate transition metals, and scavenge lipid peroxidation products; however, its role in protecting against tissue injury remains unclear. In this study, we tested the hypothesis that carnosine protects against post ischemia by augmenting HIF-1α angiogenic signaling by Fe^2+^ chelation. We found that wild type (WT) C57BL/6 mice, subjected to hind limb ischemia (HLI) and supplemented with carnosine (1g/L) in drinking water, had improved blood flow recovery and limb function, enhanced revascularization and regeneration of myocytes compared with HLI mice placed on water alone. Carnosine supplementation enhanced the bioavailability of carnosine in the ischemic limb, which was accompanied by increased expression of proton-coupled oligopeptide transporters. Consistent with our hypothesis, carnosine supplementation augmented HIF-1α and VEGF expression in the ischemic limb and the mobilization of proangiogenic Flk-1^+^/Sca-1^+^ cells into circulation. Pretreatment of murine myoblast (C2C12) cells with octyl-D-carnosine or carnosine enhanced HIF-1α protein expression, VEGF mRNA levels and VEGF release under hypoxic conditions. Similarly pretreatment of WT C57/Bl6 mice with carnosine showed enhanced blood flow in the ischemic limb following HLI surgery. In contrast, pretreatment of hypoxic C2C12 cells with methylcarcinine, a carnosine analog, lacking Fe^2+^ chelating capacity, had no effect on HIF-1α levels and VEGF release. Collectively, these data suggest that carnosine promotes post ischemic revascularization via augmentation of pro-angiogenic HIF-1α/VEGF signaling, possibly by Fe^2+^ chelation.

## Introduction

Carnosine (β-alanine-histidine) is an endogenous histidyl dipeptide, present in high concentration in the skeletal muscle (1–20 mM), heart (0.1–1 mM), and brain (1–2 mM). Among the naturally occurring histidyl dipeptides, carnosine is the most abundant in the human skeletal muscle, whereas its natural analogs such as anserine (β-alanine-N-π-histidine) and balenine (β-alanine-N-τ histidine) are found in other avian and mammalian species (Boldyrev et al., 2013). These dipeptides are multifunctional which have the capacities to chelate transition metals, such as Cu^2+^ and Fe^2+^, sequester lipid peroxidation products, such as 4-hydroxy *trans*-2-nonenal (HNE), act as antioxidants, and buffer intracellular pH (Hipkiss, 2009; Baba et al., 2013; Boldyrev et al., 2013; Zhao et al., 2018; Ihara et al., 2019). In mammalian tissues, carnosine synthesis is catalyzed by the ATP ligase enzyme – carnosine synthetase (ATPGD1) (Drozak et al., 2010), and its homeostasis within the muscle is maintained by a complex interplay between transporters (PHT, PEPT2, and TAUT) (Everaert et al., 2013a) and hydrolases (CNDP1 and 2) (Teufel et al., 2003). Previous work suggests that carnosine increases skeletal muscle and myocardial contractility (Zaloga et al., 1997; Everaert et al., 2013b), improves post ischemic recovery in both rat and mice hearts (Lee et al., 2000) and plays a neuroprotective role in focal cerebral ischemia (Bae et al., 2013; Baek et al., 2014). Although skeletal muscle is the major carnosine depot, the role of the dipeptide in preventing skeletal muscle injury has not been studied, and it is unclear whether carnosine affects ischemic damage or post injury events underlying wound healing and tissue angiogenesis.

Recurrent, unabated ischemia is the underlying cause of tissue loss in peripheral arterial disease (PAD) or peripheral vascular disease (PVD). Even though the exact mechanisms underlying ischemic injury in the muscle are not completely understood, hypoxia-inducible factor-1 alpha (HIF-1α) has emerged as an attractive target to enhance post ischemic angiogenesis. HIF-1α is a transcription factor, ubiquitously expressed in most tissues of mice, humans and rats. It controls the expression of a wide repertoire of genes that promotes angiogenesis, glycolysis, cell differentiation and proliferation (Gradin et al., 1996; Wiener et al., 1996). The angiogenic signaling pathway mediated by HIF-1α activity is tightly regulated under normoxic and hypoxic conditions. Under normoxic conditions HIF-1α is hydroxylated, ubiquitinated, and degraded by prolyl hydroxlase domain proteins (PHDs), von Hippel-Lindau tumor suppressor (pVHL) and 26S proteasome, respectively, whereas during hypoxia PHDs are inactivated, HIF-1α is stabilized and translocated to nucleus. Inside the nucleus, it binds with its nuclear partner HIF-1β, and via interaction with hypoxia response elements (HRE) transcribes proangiogenic genes such as VEGF (Epstein et al., 2001; Semenza, 2003; Berra et al., 2006; Sahara et al., 2010). Several animal studies had shown that genetic delivery of HIF-1α or targeting HIF-1α activity enhances angiogenic response and blood flow in ischemic tissue. The increase in angiogenic response is largely attributed to VEGF and the mobilization of angiogenic cells (Vincent et al., 2000; Warnecke et al., 2003; Knowles et al., 2004; Milkiewicz et al., 2004; Bosch-Marce et al., 2007; Rey et al., 2009). However, clinical trials with the intramuscular administration of HIF-1α gene has been found to be largely ineffective for patients with intermittent claudication, which was attributed to the inability of viruses to diffuse across ischemic tissue (Creager et al., 2011). Because HIF-1α activity is regulated by PHDs, which requires oxygen, 2-oxoglutarate, and iron (Fe^2+^) as cofactors (Bruick and McKnight, 2001; Ivan et al., 2001; Jaakkola et al., 2001), several pharmacological inhibitors and Fe^2+^ chelators are available to inhibit PHDs activity and enhance revascularization (Wang and Semenza, 1993; Ikeda et al., 2011), but the toxicity associated with the metal chelators and inhibitors, limits their clinical applications (Warnecke et al., 2003). Nevertheless, the HIF-1α angiogenic signaling pathway is considered a significant target to enhance angiogenesis. Therefore, non-toxic therapies that could readily diffuse across the ischemic tissues and stimulate HIF-1α post ischemic angiogenesis are needed.

Given the evidence that carnosine is a safe food constituent, which exhibits the ability to chelate Fe^2+^ (Baba et al., 2013; Canabady-Rochelle et al., 2015; Zhao et al., 2018) that inactivates PHDs, and enhances HIF-1α-mediated angiogenic response (Milkiewicz et al., 2004), we designed this study to investigate whether oral intake of carnosine could enhance post ischemic angiogenic responses in mice subjected to HLI. Our results showed that carnosine supplementation during HLI accelerated blood flow recovery and revascularization in the ischemic skeletal muscle by increasing HIF-1α/VEGF levels in the ischemic muscle. These findings provide a new insight into the physiological role of carnosine and suggest safe therapeutic intervention that might be beneficial in enhancing post ischemic revascularization.

## Materials and Methods

### Animals and Reagents

Male C57BL6J mice (16–18 weeks old) were obtained from Jackson Laboratory (Bar Harbor, ME). They had access to chow *ad libitum* and were placed on a 12 h:12 h light/dark cycle. All protocols and procedures were approved by the Institutional Animal Care and Use Committee of the University of Louisville. The following critical reagents were purchased from commercial vendors: carnosine, hematoxylin, and eosin dyes, histamine dihydrochloride, EDC [*N*-(3-Dimethylaminopropyl)-*N*′-ethylcarbodiimide], and *N*-methylmorpholine (Sigma-Aldrich); dry DCM and dry DMF (Acros Organics), microfil dye (Flow Tech Inc.); isolectin antibody (Molecular Probes); antibodies to PEPT2/SCL15A2 (ab 186999; abcam); HIF-1α (Sc-13515; Santa Cruz), and PHT1/SLC15A4 (NB1-87279; Novus biological), HDAC (05-814; Millipore), and VEGF (Sc-7269; Santa Cruz).

### Hind Limb Ischemia (HLI) and Laser Doppler Perfusion Imaging

Hind limb ischemia was induced as described previously (Abplanalp et al., 2016; Zhang M.J. et al., 2016). Male mice were anesthetized by isoflurane inhalation (3% isoflurane mixed with 100% O_2_) and anesthesia was maintained by continued isoflurane inhalation (1–2% isoflurane). The left groin area was shaved, a small incision was made through the skin, and the femoral artery and vein were exposed. Sterile 7.0 sutures were threaded gently under and around the femoral artery and vein, and knotted for permanent ligation around both the vessels -2 mm apart. Skin was closed using sterile nylon 6.0 sutures and tissue adhesive sealant. Buprenorphine (0.5 mg/kg; i.p.) was given twice within 24 h post surgery. Sham surgery was conducted in the same manner, except that the blood vessels were not ligated. Following sham and HLI surgeries mice were divided into two groups and treated with carnosine (1 g/L) or water alone for 21 days. For pretreatment studies, the mice were supplemented with carnosine (1 g/L) or water alone for 7 days and subjected to HLI surgery. After 7, 14-, and 21-days of surgery, mice were anesthetized as above and laser Doppler perfusion Images, (LDPI; using either Perimed or MoorLDI2; Moor 40 Instruments) were acquired to assess blood flow in both ischemic and non-ischemic feet and hind limbs. After acquisition, color-binned images of mouse limbs were imported into Image J, and areas of blue, green and red in the region of interest (ROI) spanning the entire hind limb or feet were quantified. The value for the ischemic limb was then normalized to the non-ischemic limb to calculate % recovery of blood perfusion for each mouse. Two images were taken and analyzed for every mouse, and return of blood flow (recovery) to ischemic leg was quantified as % blood flow in the non-ischemic leg (Abplanalp et al., 2016; Zhang M.J. et al., 2016).

### Vascular Casting and Micro-Computed Tomography (MicroCT)

On postoperative day 21, the limb vasculature was quantified and visualized using vascular casting, followed by microcomputed tomography analysis (Simons et al., 2015; Zhang M.J. et al., 2016). For this procedure, the mice were injected with 1000 U/mL; IP) heparin to prevent blood coagulation and sedated with pentobarbital (50 mg/kg). An incision was made in the thoracic cavity to expose the heart and then cannulated with a 15G needle and systemically perfused at a constant flow rate of 5 mL/min with the vasodilation solution (100 μmol/L adenosine (10 mL), followed by 10 μmol/L sodium nitroprusside and then bovine serum albumin (0.05% W/V). Undiluted Microfil vascular casting agent was mixed with 10% volume of curing agent and perfused systemically. Casting was allowed to polymerize at room temperature and the entire hindlimb was deskinned. To obtain whole-mount images, deskinned limbs were placed in increasing concentration of glycerol (40, 60, 80, and 100%) for 24 h. Whole mount images were obtained by imaging with a digital CCD camera (Nikon). For MicroCT analysis, deskinned limbs were fixed in formalin for 24 h, followed by decalcification with Cal EX II for 48 h. Samples were stored in 70% ethanol until further imaging. MicroCT imaging for the entire hindlimb was carried out on a MicroCT (Siemens) using following parameters; 80 kVp, 200 μA and 2 × 2 binning to obtain a pixel resolution of 1024 × 1024 with 34 μM voxel sizes. After setting appropriate thresholds for soft tissue elimination, blood vessel volumes were quantified using the Analyze software.

### Immunofluorescence

Mice were euthanized after 7 days of HLI, and hamstring skeletal muscle from ischemic limbs was collected, formalin-fixed, and paraffin-embedded. Thick sections (5 μm thick) of the muscle were mounted on glass slides and stained with hematoxylin and eosin staining (H&E) for immunofluorescence. The sections were viewed on an Olympus IOM microscope and imaged with a SPOT camera using SPOT advanced image-capture software. Each image was embedded with a SPOT software-generated calibration line (e.g., 1000 μm) stamp for subsequent image analysis. The number of myocytes with centrally located nuclei indicates muscle regeneration which was used to assess the extent of limb damage and recovery with carnosine treatment (Ciciliot and Schiaffino, 2010). Five to ten low power fields were examined in each section and the total number of myocytes with centrally located nuclei was determined and expressed as a percent (%) of the total myocytes per field (Zhang M.J. et al., 2016). Sections were also stained with fluorescein-conjugated isolectin (FITC-Griffonia (Bandeiraea) Simplicifolia Lectin I, Vector Labs) and 4′,6-diamidino-2-phenylindole (DAPI, nuclear counterstain) and images acquired with a fluorescent microscope. Analysis of staining was performed using free software NIH ImageJ (ver. 1.45 s). Each digital photomicrograph (up to three different sections per slide) was analyzed as reported previously (Abplanalp et al., 2016).

### Physical Activity After Hindlimb Ischemia

Limb function, i.e., physical activity of the HLI mice was assessed by using metabolic chambers (TSE PhenoMaster system; Bad Homberg Germany). Mice were kept in the chamber overnight and the locomotor movements (fine and ambulatory) were recorded and used as a measure of limb function, along with oxygen consumption and CO_2_ production.

### Measurement of Histidyl Dipeptides and Carnosine-Aldehyde Conjugates

Levels of carnosine in the hamstring muscle of mice were measured as described previously (Baba et al., 2013). Briefly, the mice were subjected to 3 days of HLI, and the muscle was homogenized in a lysis buffer containing PBS, protease inhibitor cocktail, and 200 μM Tyr-His internal standard (IS). The homogenates were sonicated and centrifuged at 16000 × *g*. Following centrifugation, the supernatant was de-proteinated with 70% perchloric acid and centrifuged 16000 × * g*. The resulting supernatant was neutralized with 750 mM ammonium hydrochloride and diluted 1:1 with the mobile phase (90% water: 9.9% acetonitrile and 0.1% heptaflurobutyric acid). Samples were injected into HP 1100 LC and separated on a Polar RP column. Histidyl dipeptides were detected by using Micromass Quattro LC/MS/MS system (Blancquaert et al., 2016). The data for histidyl dipeptides were acquired by monitoring the following transitions 226.95→110.22 (carnosine), and 319→110.22 (Tyr-His, internal standard). The peptides were quantified using the peak area ratio of histidyl-dipeptide and IS. MassLynx mass spectrometry software from Waters was used for analysis. To determine the uptake of β-alanine and synthesis of carnosine in the C2C12 cells and human umbilical vein endothelial cells (HUVECs), the cells were treated with different concentrations of β-alanine (0.1–10 mM) for 24 h. To determine the uptake of ODC and methylcarcinine, C2C12 cells were treated with varying concentration of dipeptide (0.1–1 mM) for 6–12 h. Cells were lysed in PBS with protease inhibitor and IS as described above. For ODC the 321.95→110.22 *m/z*, methylcarcinine 223→110.22 *m/z*, and β-alanine 90→30 *m/z* transitions were monitored. Dipeptide levels in cells were normalized to protein in the precipitate, measured by precipitation Lowry protein assay (Peterson, 1977).

### Western Blot Analysis

Hamstring muscles were isolated from the mice 3 days after HLI or sham surgeries. The tissue collected from the contralateral and ischemic limb, distal to the site of surgery was homogenized in RIPA buffer (20 mM Tris–HCl pH 7.5, 150 mM NaCl, 1 mM EDTA, 1 mM EGTA, 1% NP-40) and separated by SDS-PAGE. Immunoblots were analyzed using anti-HIF1α, anti-VEGF, anti-PEPT2, anti-PHT1, and anti-HDAC antibodies. Membranes were developed using HRP substrate (ECL plus form Pierce) and scanned using a Typhoon Bioimager (GE Healthcare).

### Quantitative RT-PCR Analysis

Total RNA was isolated from hamstring muscle distal to the site of surgery following 3 days of HLI and sham surgeries using Qiagen Fibrous Tissue RNA isolation kit. Expression of the genes encoding *Atpgd1, Cndp2, Pht1, TauT, Vegf*, and *Pept2* were determined using quantitative RT-PCR and primers described previously by Everaert et al. (2013a) (Table 1). Results were normalized to HPRT1 and expressed according to the comparative C_t_ method that compares the C_t_ values of the gene of interest and sham as an internal control gene (Livak and Schmittgen, 2001). Measurements were made using Prism 7900 HT (Applied Biosystem).

**Table 1:** Primers for qRT-PCR.

Gene	Forward primer (5′-3′)	Reverse primer (5′-3′)
Carnosine synthase (*ATPGD*1)	TGA_TAG-GCC-CCT-ACT-GAG-TAA-GGT	TCA-GTG-TCC-TTG-GCA-GGG-TAT
Carnosinase 2 (*CNDP2*)	GGA-GAT-ACC-ACT-TCC-TCC-CAT-TC	CGT-CCA-GGT-GCC-CGT-AAA-T
Peptide transporter (*PEPT2)*	TGG-CTG-GGA-AAA-TTC-AAG-ACA	ATG-GCA-CCC-AAA-GAC-TTG-AAT-AC
Carnosine histidine transporter (*PHT1*)	CAT-GTG-TCC-GTG-GTG-ATT-GAG	GCG-TGG-TGT-AAC-TGC-CAA-TCT
β-alanine transporter (*Taut*)	TGG-CCG-ACA-GCA-TTC-CA	GCC-TTC-TCT-AAG-GTG-CCT-TCC-T


### *In vitro* Hypoxia of Murine Myoblasts

The C2C12 cells, maintained in DMEM supplemented with 10% fetal bovine serum (FBS) and 0.1% penicillin/streptomycin, were allowed to differentiate by replacing the FBS with 2% horse serum. Differentiated cells were pretreated with ODC (50–500 μM), carnosine (1 mM) or methylcarcinine (MC, 1 mM) for 8–12 h. To simulate ischemia, differentiated C2C12 cells were subjected to hypoxia for 5, 10, 15, 30 min, and 4 h in Esumi lethal ischemia medium for glucose and nutrient deprivation containing mM: 117 NaCl, 0.9 CaCl_2_, 12 KCl, 0.49 MgCl_2_, 4 HEPES, 20 sodium lactate and 5.6 L-glucose; pH 7.4 (Ngoh et al., 2008). For hypoxia, the C2C12 cells were placed in a sealed humidified chamber (Billups-Rothenberg Inc.) replacing oxygen with 95% N_2_ and 5% CO_2_. Cells were harvested in lysis buffer for Western blot and quantitative PCR analysis.

### Metal Chelating Capacity

The iron II (Fe^2+^) chelating ability of carnosine and methylcarcinine was monitored by measuring the formation of ferrous-ferrozine complex as described previously (Canabady-Rochelle et al., 2015; Zhao et al., 2018). Briefly, 2 mM ferrous chloride (FeCl_2_) solution was added to different dipeptide solutions in pure water. After 3 min of incubation at room temperature, the chelation reaction was inhibited by the addition of 5 mM ferrozine solution. The absorbance was measured at 560 nm after 10 min. EDTA was used as a reference chelator and the metal-chelating capacity of dipeptides was calculated in comparison to EDTA. Metal chelating capacity was measured as a decrease in the absorbance of ferrous-ferrozine complex and reported as follows:

(a) $$\mathrm{Iron}\;\mathrm{chelating}\;\mathrm{capacity}\;(\%)\;=\;(A_{0}-A_{s}/A_{0})\;\times \;100$$

A_0_ is the blank absorbance and A_s_ is the absorbance of the sample dipeptide. The line slope obtained for each dipeptide was compared with EDTA to determine the EDTA equivalent chelating capacity expressed as μmol EDTA equivalent. EDTA equivalent chelating capacity for each dipeptide was calculated as follows:

(b) $$\mathrm{EDTA}\;\mathrm{equivalent}\;\mathrm{chelating}\;\mathrm{capacity}\;=\;a_{s}/a_{o}$$

where a_s_ is the line slope of dipeptide for chelating activity, plotted as iron chelating capacity (percent) vs. concentration (μM), a_0_ is the line slope of EDTA for chelating activity, plotted iron chelating capacity (percentage) vs. concentration (μM).

### Flow Cytometry Analysis

To identify the cell population that contributes to blood flow recovery, flow cytometry analysis of peripheral blood was performed 3 and 7 days after HLI or sham surgery as previously described (Wheat et al., 2011). Following euthanasia, blood was collected by cardiac puncture. Blood samples were lysed, blocked by Fc, and washed with 1% BSA in PBS. Samples were stained for leukocyte markers using a cocktail of the following antibodies: CCR2, CD11b, F4/80, Ly6C^+^, 7/4, CD19, CD4, CD8, Flk-1^+^, and Sca1^+^. Samples were stained with antibody cocktail for 30 min, washed with PBS containing 1% BSA. Different cell populations were identified using a BD LSR II flow cytometer and Flowjo software.

To determine monocyte infiltration in muscle, the hamstring muscle of ischemic and contralateral limbs were excised at day 3 of surgery, weighed, and digested in 0.2% Type II collagenase at 37°C for 1 h. The digested tissue was passed through a 50-μm cell strainer, washed with 1% BSA in PBS, Fc blocked and then stained with anti-F4/80, as described previously (Capoccia et al., 2008).

### Data Analysis and Statistics

All experimental results are mean ± SEM. For studies involving 2 groups, an unpaired Student *t*-test was used for analysis and for studies involving more than 2 groups, a one-way ANOVA or repeated measures ANOVA was performed followed with Bonferroni correction. All statistical analyses were performed using the GraphPad analysis software. A *p* value < 0.05 was considered statistically significant.

## Results

### Carnosine Treatment Accelerates Blood Flow Recovery and Tissue Regeneration Following Hind Limb Ischemia (HLI)

**FIGURE 1:**
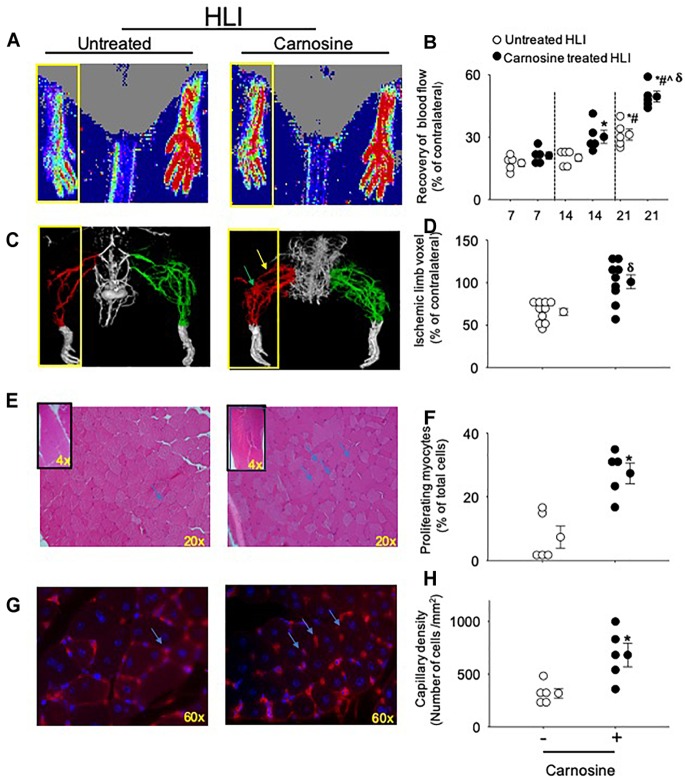
Carnosine supplementation enhances blood flow recovery and tissue regeneration during HLI. **(A)** Representative Laser Doppler perfusion images of foot perfusion in mice treated with carnosine (1 mg/mL) or water alone after 21 days of HLI. Yellow box indicates the ischemic paw. **(B)** Tissue perfusion is presented as percent (%) of blood flow in the non-ischemic paw. **(C)** Representative micro-CT images showing limb vasculature on day 21 post HLI in mice that were treated with carnosine or water alone. Arteriogenesis and angiogenesis were determined by Microfil perfusion analysis. The yellow box indicates the area of interest identifying the cork-screw like collateral vessels indicated by green arrow and neo-angiogenesis indicated by blue arrows. **(D)** Quantitative evaluation of vascular volume by 3D micro-CT analysis. **(E)** Representative images (20×) of Hematoxylin and Eosin (H&E) staining in skeletal muscle after 7 days of HLI surgery in carnosine and un-treated mice. Inset shows the representative images (4×) of H&E staining and **(F)** quantification of myocytes with centrally located nuclei. **(G)** Isolectin (60 × images) staining of hind limb sections following 7 days of recovery from HLI surgery in carnosine treated and un-treated mice and, **(H)** capillary density is presented as ratio of isolectin B4 (+) cells and DAPI (+) cells/mm^2^. Data are mean ± SEM, *n* = 5–10 mice per group, ^∗^*p* < 0.05 vs. 7 days of untreated HLI mice, ^#^*p* < 0.05 vs. 14 days of untreated HLI, ˆ*p* < 0.05 vs. 14 days of carnosine treated HLI, ^δ^*p* vs. 21 days of untreated HLI.

We first tested whether carnosine treatment could improve blood flow recovery post HLI surgery. For these experiments, wild type (WT) male C57BL/6 mice were subjected to HLI and divided into two groups that were placed on drinking water without or with carnosine (1 mg/mL) for 21 days. Revascularization in the hind paw was assessed serially using LDPI. Blood perfusion was quantified as a percentage of blood flow in the ischemic to non-ischemic hind paw. The results of these analyses indicated that the perfusion ratio at day 7 of the HLI surgery was similar between the untreated and carnosine treated HLI mice (untreated: 18 ± 2% and carnosine: 21 ± 2%). The perfusion ratio enhanced at day 14 in the carnosine-treated (30 ± 3%, *p* < 0.05) vs. untreated HLI mice following 7 days of surgery (untreated: 18 ± 2%). Blood flow was increased in the untreated HLI mice at day 21 of the surgery compared with the untreated HLI at day 7 of the surgery (untreated 7 days HLI: 18 ± 2% vs. untreated 21 days HLI: 31 ± 3%). However, the blood flow in the carnosine treated mice at day 21 of the HLI surgery (49 ± 3%, *p* < 0.05) was significantly enhanced compared with the untreated HLI mice following 14 (20 ± 2%) and 21 days of surgery (31 ± 3%, Figure 1A,B).

We next assessed the development of collateral blood vessels in carnosine-fed mice after HLI. For this, anesthetized mice were whole-body perfused with Microfil at day 21 post HLI surgery, and the limb vasculature was quantified by 3D microCT. Whole mount images of Microfil-casted limbs indicated that carnosine-treated mice had a higher proportion of more developed collateral arteries and an increased number of cork screw–like collateral blood vessels after HLI compared with untreated HLI mice. Quantitative analysis of the angiographic score (vascular volume, density, and connectivity), which was calculated as a ratio of ischemic to non-ischemic (contralateral) hind limb, was significantly increased by carnosine intervention (*p* < 0.05) compared with untreated HLI mice (Figure 1C,D). To determine whether carnosine treatment improves tissue regeneration, we performed Hematoxylin and Eosin (H&E) staining of the ischemic muscle at day 7 post HLI. Histological analyses of the skeletal muscle showed that carnosine treatment significantly increased myocyte regeneration, which was characterized by centrally located nuclei and regular polygonal shaped myofibers (Figure 1E,F; Zhang M.J. et al., 2016).

**FIGURE 2:**
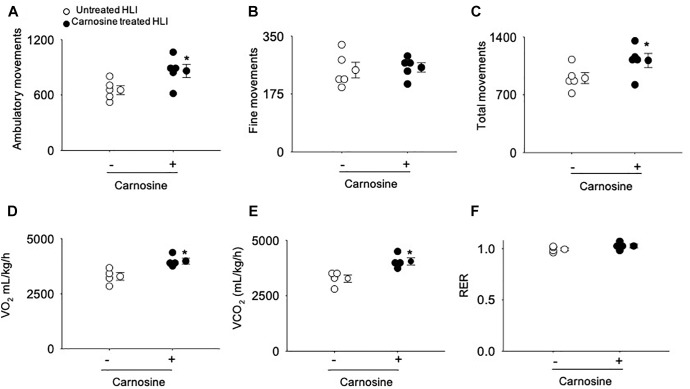
Carnosine supplementation increases ambulatory movement in HLI mice. Quantitative analysis of **(A)** ambulatory movements, **(B)** fine movements, **(C)** total movements **(D)** VO_2_, **(E)** VCO_2_ and **(F)** respiratory exchange ratio (RER) measured in metabolic cages after 14 days of HLI. Data are mean ± SEM, *n* = 5 mice per group, ^∗^*p* < 0.05 vs. untreated HLI mice.

To quantify angiogenesis of the ischemic limb, we assessed the number of isolectin B4 (+) blood vessels at day 7 after HLI surgery. Analysis of immunofluorescent staining of the ischemic limb showed that the capillary density (number of cells/mm^2^) in the ischemic muscle of carnosine-treated mice was 2–3-fold higher compared with untreated HLI mice (Figure 1G,H). Finally, to determine whether the enhanced revascularization and recovery of blood flow by carnosine intervention contributes to functional changes, locomotor activity was measured in the mice that were placed in metabolic cages at 14 days after HLI induction. Both average ambulatory (gross) and total nighttime movements of carnosine-treated HLI mice were significantly increased (ambulatory: 860 ± 80/12 h, total: 1117 ± 95/12 h; *p* < 0.05) compared with the untreated HLI mice (ambulatory 654 ± 54/12 h, total: 901 ± 74/12 h), whereas no change was observed in the number of fine movements (Figure 2A–C). In addition, the nighttime rates of oxygen consumption and CO_2_ production in the carnosine-treated mice also were increased compared with untreated HLI mice (VO_2_: 3977 ± 133 vs. 3283 ± 175 mL/kg/h; VCO_2_: 4050 ± 320 vs. 3278 ± 165 mL/kg/h), whereas the respiratory exchange ratio (RER) remained unchanged (Figure 2D–F). Taken together, these data suggest that carnosine feeding during HLI accelerates blood flow recovery and post ischemic revascularization facilitating a more rapid return of locomotor activity.

### Carnosine Transport Is Enhanced in Ischemic Skeletal Muscle

**FIGURE 3:**
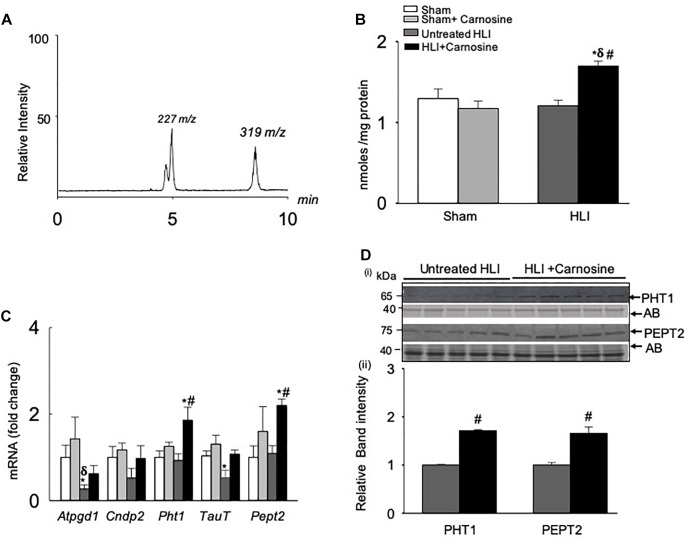
Carnosine transport is enhanced in the hamstring skeletal muscle during hind limb ischemia (HLI). **(A)** Representative *LC/MS/MS* spectra of carnosine (*m/z* 227) and internal standard (*m/z* 319) detected in the hamstring skeletal muscle. **(B)** Quantitative analysis of carnosine in the hamstring skeletal muscle normalized to total protein. **(C)** qRT PCR shows the mRNA levels of *Atpgd1, Cndp2, Pht1, TauT, and Pept2* in the hamstring skeletal muscle after 3 days of sham and HLI surgeries without and with carnosine. **(D)** (i) Representative Western blots of PHT1 and PEPT2 normalized to amido-black (AB) in the ischemic limb of carnosine treated and untreated mice 3 days after HLI, (ii) lower panel shows the quantitative analysis for PHT1 and PEPT2. Data are mean ± SEM, *n* = 3–5 mice in each group, ^∗^*p* < 0.05 vs. sham, ^δ^*p* < 0.05 vs. sham + carnosine, ^#^*p* < 0.05 vs. untreated HLI.

To determine the mechanism by which carnosine affects the recovery from ischemic injury, we first examined whether carnosine supplementation in drinking water increases the carnosine levels in the ischemic skeletal muscle. For these experiments, adult C57BL/6 mice were subjected to either sham or HLI surgery and then randomly divided into two groups which were placed on either drinking water alone or drinking water with carnosine (1 mg/mL) for 3 days. The levels of carnosine in the hamstring skeletal muscle of carnosine-treated HLI mice, measured by *LC/MS/MS*, were significantly increased compared with untreated HLI mice (HLI+carnosine:1.69 ± 0.06 vs. untreated HLI: 1.21 ± 0.06 nmol/mg protein, *p* < 0.05). Interestingly, no change in carnosine levels was observed in the hamstring skeletal muscle of carnosine-treated vs. untreated sham-operated mice (sham+carnosine 1.29 ± 0.12 vs. untreated sham 1.17 ± 0.09 nmol/mg protein) suggesting that the carnosine bioavailability is enhanced only in the ischemic muscle of the mice subjected to HLI (Figure 3A,B).

To determine the mechanism by which carnosine feeding increased carnosine levels in the ischemic skeletal muscle, gene expression of the enzymes and transporters that regulate carnosine homeostasis in the skeletal muscle (Everaert et al., 2013a) were measured in the hamstring skeletal muscle of the sham and HLI operated mice. Gene expression of the enzyme *Atpgd1*, which synthesizes carnosine (Drozak et al., 2010), was decreased ≈2-fold in the untreated-HLI mice compared with the sham-operated untreated and carnosine-treated mice. Although *Atpgd1* levels appeared lower in the carnosine-fed HLI mice compared with sham-operated mice, but the gene expression did not reach statistical significance. Expression of the carnosine transporters *PHT1* and *PEPT2* in the ischemic skeletal muscle of carnosine-treated HLI mice was increased ≈2-fold compared with the sham and untreated HLI-operated mice (Figure 3C). Furthermore, mRNA levels of the Taut, that were decreased during ischemia were normalized by carnosine treatment in the ischemic muscle. No change was observed in the mRNA levels of carnosine hydrolyzing enzyme *CNDP2* in any treatment (Figure 3C). For validation of mRNA levels, protein lysates from the ischemic tissue of carnosine-treated and untreated HLI mice were probed with PHT1 and PEPT2 antibodies. Immunoblot analysis showed that the levels of PHT1 and PEPT2 were increased in the hamstring skeletal muscle of carnosine-treated HLI mice compared with the untreated HLI mice (Figure 3D). Taken together, these findings suggest that carnosine transport into ischemic skeletal muscle was enhanced by induction of carnosine transporters.

### Enhanced Carnosine Transport Augments HIF-1α and VEGF Expression *in vivo*

**FIGURE 4:**
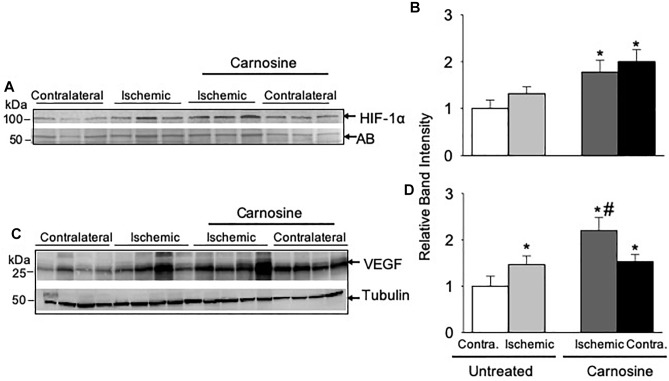
HIF-1α mediated angiogenic signaling is enhanced by carnosine supplementation in the ischemic limb. Representative Western blots of **(A)** HIF-1α and **(C)** VEGF in the contralateral (contra.) and ischemic hamstring muscles isolated from the mice 3 days after HLI surgery. Mice were placed on drinking water with or without carnosine (1 mg/mL) as indicated. Quantitative analysis of **(B)** HIF-1α and **(D)** VEGF normalized to amido-black (AB) and tubulin. Data are mean ± SEM, *n* = 3–4 mice in each group, ^∗^*p* < 0.05 vs. untreated contralateral HLI, ^#^*p* < 0.05 vs. untreated ischemic limb from HLI mice.

Given our observation that carnosine supplementation enhanced blood flow, we next examined the underlying molecular mechanisms by which carnosine increases revascularization. Numerous studies have shown that HIF-1α is the master regulator of angiogenic signaling pathway (Forsythe et al., 1996; Semenza, 2003; Sarkar et al., 2009). To determine whether the increased transport of carnosine to ischemic limb enhances HIF-1α mediated angiogenic signaling, we examined the expression of HIF-1α and VEGF in the hamstring muscle of the contralateral and the ischemic limb of the carnosine treated and untreated mice following 3-days after surgery. As shown in Figure 4A,B the protein expression of HIF-1α in the hamstring muscle of the ischemic and the contralateral limbs of the carnosine-treated HLI mice were increased up to 1–2-fold, when compared with the contralateral limb of un-treated mice. To further examine whether the increase in HIF-1α expression is transcriptionally active, we examined VEGF expression in the hamstring muscle. We found that VEGF expression was increased in the ischemic limb of the un-treated and the contralateral limb of the carnosine treated mice compared with the contralateral limb of the un-treated mice, which was further augmented by carnosine treatment in the ischemic limb (Figure 4C,D). Taken together, these results suggest that the greater bioavailability of carnosine during ischemia enhances HIF-1α mediated angiogenic signaling pathway.

### Carnosine Enhances Angiogenic Signaling Pathway by Targeting Skeletal Myoblasts

**FIGURE 5:**
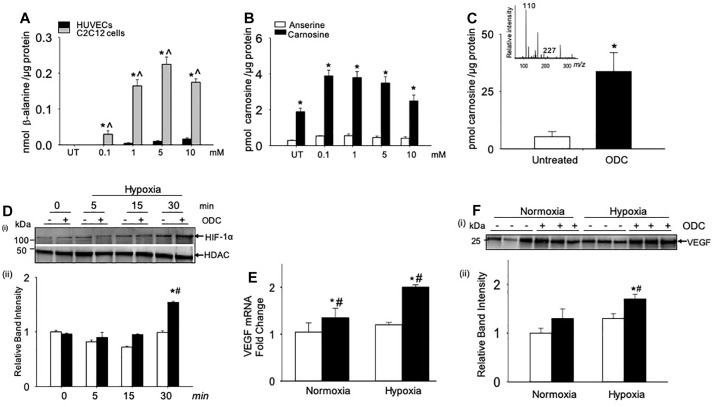
Angiogenic signaling pathway is enhanced by carnosine within the skeletal muscle myoblasts. **(A)** Quantitative analysis of β-alanine by LC/MS in human endothelial cells (HUVECs) and skeletal muscle myoblasts (C2C12 cells), which were incubated with different concentrations of β-alanine (0.1–10 mM) for 24 h. **(B)** Levels of carnosine and anserine measured by LC/MS in C2C12 cells either untreated (UT) or treated with β-alanine for 24 h. **(C)** Quantitative analysis of octyl D carnosine, ODC (100 μM) in C2C12 cells incubated with ODC for 6–12 h measured by *LC/MS/M*S, inset shows the fragmentation pattern of carnosine detected by monitoring ions *m/z* 227 and 110, using tyrosine-histidine (*m/z* 319) as an internal standard. **(D)** Representative Western blots of HIF-1α and HDAC in nuclear extracts that were preincubated with octyl-D carnosine (ODC;100 μM) and subjected to different durations of hypoxia (3% O_2_), (ii) lower panel shows the densitometry analysis of HIF-1α normalized to HDAC. **(E)** Quantitative analysis of VEGF mRNA levels in C2C12 cells pretreated with ODC (100 μM) that were subjected to 4 h of hypoxia. **(F)**, (i) Representative Western blots of VEGF secreted in media by C2C12 cells that were pretreated with ODC (100 μM) and subjected to 4 h of hypoxia, (ii) lower panel shows the densitometry analysis of VEGF normalized to protein in the cell lysate. Data are presented as mean ± SEM, *n* = 3–5 in each treatment, ^∗^*p* < 0.05 vs. untreated (UT) cells, ˆ*p* < 0.05 vs. HUVECs, and ^#^*p* < 0.05 vs. untreated hypoxic cells.

Given that carnosine treatment enhanced HIF-1α/VEGF levels in the ischemic skeletal muscle, we next tested, which cells within the muscle could be involved in enhancing angiogenesis and response to carnosine treatment. For these experiments, we treated the murine myoblasts (C2C12) cells and HUVECs with different concentrations of β-alanine (0.1–10 mM) for 24 h. Since β-alanine is the rate limiting amino acid for carnosine synthesis and transported by the transporters similar to carnosine (Everaert et al., 2013a; Hoetker et al., 2018), we measured the β-alanine uptake and the carnosine synthesis in the β-alanine treated C2C12 and HUVEC cells by LC/MS. Our results showed that the uptake of β-alanine in the HUVECs was approximately 10–20-fold lower compared with the C2C12 cells (Figure 5A). Significantly, our results showed that the carnosine synthesis was increased in the β-alanine treated C2C12 cells compared with the non-treated C2C12 cells (Figure 5B), whereas in HUVECs the untreated or β-alanine treated cells, carnosine was undetectable suggesting that the transporters and the enzymes involved in transport and synthesis of carnosine are active in the skeletal muscle myoblasts compared with the endothelial cells.

Given that the myoblasts are the major contributors of carnosine synthesis within the skeletal muscle, we next examined whether the direct treatment of murine myoblasts (C2C12 cells) with carnosine could regulate HIF-1α/VEGF signaling under hypoxic conditions. For these experiments, murine myoblasts were preincubated with a membrane permeable analog of carnosine, octyl-D-carnosine (ODC; 100 μM), for 10–12 h. Treatment with ODC increased the intracellular level of carnosine in C2C12 cells by 2–3-fold compared with the naïve, untreated cells (Figure 5C). To determine the effect of carnosine on HIF-1α induction under ischemic conditions, ODC preloaded C2C12 cells were subjected to different durations of hypoxia (0–30 min). After 30 min of hypoxia, the accumulation of HIF-1α in the nuclear fraction of ODC pretreated cells was significantly increased compared with the naïve cells maintained under hypoxic or normoxic conditions (Figure 5D). To examine whether the carnosine-induced HIF-1α was transcriptionally active, we quantified the gene expression of VEGF and found that the preincubation of C2C12 cells with ODC enhanced the levels of VEGF mRNA after 4 h of hypoxia when compared with the untreated hypoxic and normoxic cells (Figure 5E). Furthermore, the levels of VEGF secreted in the medium were higher in the ODC-treated cells compared with the untreated cells subjected to hypoxia (Figure 5F). Collectively, these results show that increased transport of carnosine into skeletal muscle myoblasts may potentiate HIF-1α/VEGF-mediated angiogenesis during ischemia.

**FIGURE 6:**
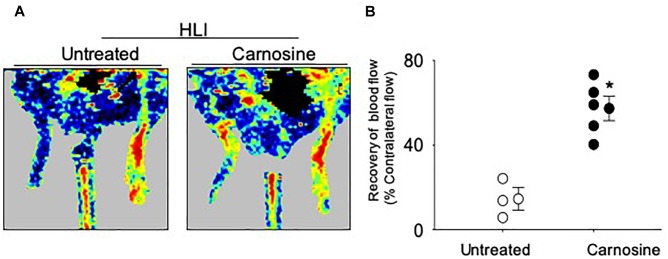
Carnosine pretreatment enhances blood flow in the ischemic limb. **(A)** Representative Doppler images of WT C57/Bl6 mice that were pretreated with carnosine (1 gm/L) or water alone for 7 days and subjected to HLI. Images were taken following 14 days of HLI surgery. **(B)** Blood perfusion is presented as a percent of the blood flow in the collateral limb. Data are mean ± SEM, *n* = 3–5 mice per group, ^∗^*p* < 0.05 vs. non-treated HLI mice.

Given that carnosine pretreatment enhanced angiogenic response in skeletal muscle myoblasts, we next examined whether the carnosine pretreatment could improve blood flow in the ischemic limb. For this, mice were pretreated with carnosine (1mg/mL for 7 days) and then subjected to HLI surgery. Carnosine treatment was continued for another 14 days. Blood perfusion measurements in the ischemic leg of carnosine pretreated mice showed significant improvement (57 ± 6%) compared with the un-treated mice (15 ± 6%; Figure 6). Collectively, these results demonstrate that carnosine pretreatment as well as intervention accelerates blood flow recovery and improves limb function after ischemia.

### Carnosine Supplementation Enhances Mobilization of Angiogenic Flk-1^+^/Sca-1^+^ Cells Into Blood

**FIGURE 7:**
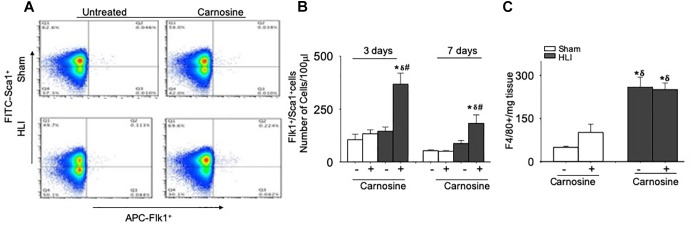
Mobilization of Flk-1^+^/Sca-1^+^ cells in the peripheral blood is augmented by carnosine feeding. **(A)** Representative flow cytometry dot blots of Flk-1^+^/Sca-1^+^ cells from peripheral blood 3 days after HLI. **(B)** Quantitative analysis of circulating levels of Flk-1^+^/Sca-1^+^ cells after 3 and 7 days of HLI surgery. **(C)** Macrophages (F4/80^+^) enumerated from skeletal muscle and analyzed 3 days after HLI. Data are number of cells per 100 μl of blood or mg of tissue. Data are mean ± SEM, *n* = 3–8 mice per group, ^∗^*p* < 0.05 vs. sham, ^δ^p < 0.05 vs. sham+carnosine, ^#^*p* < 0.05 vs. untreated HLI mice.

Previous studies have shown that VEGF release enhances the mobilization of angiogenic cells into circulation and that the revascularization of ischemic tissue is supported by several populations of angiogenic cells in the peripheral blood, such as monocytes, Flk-1^+^/Sca-1^+^ cells and T-lymphocytes (Arras et al., 1998; Isner and Losordo, 1999; Heil et al., 2004). Hence, we examined the effects of carnosine feeding on the mobilization of angiogenic cells at day 3 and 7 after surgery. Flow cytometry analysis of peripheral blood at day 3 of the surgery showed no significant difference in the number of circulating Flk-1^+^/Sca-1^+^ cells between the sham (105 ± 25 cells/100 μl blood) and untreated HLI mice (145 ± 18 cells/100 μl blood). However, the number of circulating Flk-1^+^/Sca-1^+^ cells in carnosine-treated HLI mice was significantly higher (386 ± 51 cells/100 μl blood) compared with either the sham or untreated HLI-operated mice (Figure 7A,B). The mobilization of the Flk-1^+^/Sca-1^+^ cells in carnosine-treated HLI mice was sustained and significantly higher at day 7 after the surgery (182 ± 56 cells/100 μl blood, *p* < 0.05) compared with the sham (52 ± 5 cells/100 μl blood) and HLI-operated mice (HLI: 87 ± 15 cells/100 μl blood). Although mobilization of these cells was enhanced in untreated HLI mice, this did not reach statistical significance (*p* < 0.08, Figure 7A,B). No effect of carnosine supplementation was observed on the circulating monocyte (CD11b^low/high^, LY6C^+^) levels in peripheral blood (results not shown) or the accumulation of F4/80^+^ macrophages in the ischemic limb (Figure 7C). Collectively, these results suggest that the improved recovery of blood perfusion by carnosine supplementation during HLI, may be, in part, mediated by greater mobilization of Flk-1^+^/Sca-1^+^ cells into circulation.

### Iron II (Fe^2+^) Chelation by Carnosine Regulates HIF-1α Dependent Angiogenic Signaling

**FIGURE 8:**
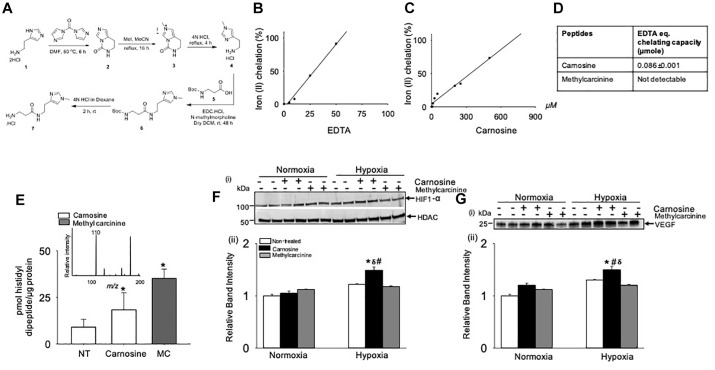
Metal chelation by carnosine is essential for hypoxia-inducible factor (HIF-1α) and vascular endothelial growth factor (VEGF) signaling pathway in hypoxic murine myoblast (C2C12) cells. **(A)** Synthesis of 3-amino-*N*-[2-(1-methyl-1*H*-imidazol-4-yl)ethyl] propanamide hydrochloride (methylcarcinine). Iron chelating capacity of **(B)** EDTA, **(C)** carnosine and **(D)** EDTA equivalent chelating activity of carnosine and methylcarcinine. **(E)** Levels of carnosine and methylcarcinine in C2C12 cells pretreated with 1 mM carnosine and methylcarcinine for 10–12 h. Inset shows the fragmentation pattern of methylcarcinine. **(F)** Representative Western blots of HIF-1α and HDAC in nuclear extract of C2C12 cells that were pretreated with carnosine (1 mM) and methylcarcinine (1 mM) and subjected to 30 min of hypoxia (3% O_2_), (ii) lower panel shows the densitometric analysis of HIF-1α normalized to HDAC. **(G)** Representative Western blots of VEGF released in media of C2C12 cells pretreated with carnosine (1 mM) and methylcarcinine (1 mM) for 10–12 h followed by 4 h of hypoxia, (ii) lower panel show the densitometric analysis of VEGF normalized to protein in the cell lysate. Data are represented as mean ± SEM, *n* = 3–4 in each treatment, ^∗^*p* < 0.05 vs. nontreated cells, ^#^*p* < 0.05 vs. non-treated hypoxic cells, ^δ^*p* < 0.05 vs. hypoxic cells pretreated with methylcarcinine.

Previous studies had shown that Fe^2+^ chelation prevents HIF-1α degradation mediated by prolyl hydroxylase domain proteins (PHDs) (Selvaraju et al., 2014). To test whether the Fe^2+^ chelating property of carnosine is involved in enhancing HIF-1α signaling in the hypoxic cell model, we synthesized a carnosine analog, methylcarcinine, in which the N^π^ of the histidyl group is methylated and lacks a carboxyl group (Supplementary Methods and Figure 8A) a functional group essential for metal binding (Torreggiani et al., 2000b). As reported previously, carnosine had Fe^2+^ chelating capacity of 0.086 ± 0.001 μmol of EDTA equivalent chelating capacity (Canabady-Rochelle et al., 2015), whereas methylcarcinine lacked the ability to chelate Fe^2+^ (Figure 8B–D). To assess the effect of methylcarcinine on HIF-1α levels, we preincubated the C2C12 cells with either carnosine (1 mM) or methylcarcinine (1 mM) for 12 h and determined the uptake of carnosine and methylcarcinine by LC/MS/MS, which was comparable between the treatments (Figure 8E). Similar to our observations with ODC treatment, we found that carnosine pretreatment increased HIF-1α expression in the hypoxic cells compared with the non-treated normoxic and hypoxic cells, whereas HIF-1α levels in the hypoxic C2C12 cells preloaded with methylcarcinine remained unchanged (Figure 8F). Furthermore, we found that the VEGF release in the culture media of carnosine-treated cells was increased compared with the non-treated normoxic and hypoxic cells, whereas in methylcarcinine-treated hypoxic cells, VEGF release remained unchanged (Figure 8G). Collectively, these observations suggest that the ability of carnosine to enhance HIF-1α/VEGF signaling axis may be attributed to its Fe^2+^ chelating property.

## Discussion

The major finding of this study is that the oral supplementation with the histidyl dipeptide – carnosine after and prior to the HLI surgery accelerates blood flow recovery in the ischemic limb of mice. We found that the expression of ATPGD1 was decreased in the ischemic limb and exogenous supplementation of carnosine enhanced carnosine transport accompanied by the induction of carnosine transporters to maintain carnosine homeostasis during ischemic injury. Revascularization by carnosine feeding was coupled with the enhanced expression of HIF-1α and VEGF in the ischemic limb and increased mobilization of pro-angiogenic Flk-1^+^/Sca-1^+^ cells into circulation. Furthermore, octyl-D-carnosine or carnosine pretreatment potentiates HIF-1α/VEGF signaling, whereas pretreatment with methylcarcinine, a histidyl dipeptide lacking Fe^2+^ chelation, is ineffective on HIF-1α/VEGF signaling in hypoxic murine myoblasts. Collectively, these results suggest that carnosine supplementation enhances post ischemic angiogenesis and minimizes ischemic injury by potentiating HIF-1α/VEGF signaling via Fe^2+^ chelation.

Carnosine is present in high concentration in muscle and a readily available food constituent. Because it has a pKa value (6.8–7.1), which is close to the physiological pH, it is believed that high levels of carnosine ensure continued glycolysis during exercise by buffering intracellular proton concentrations (Parkhouse and McKenzie, 1984; Parkhouse et al., 1985; Boldyrev et al., 2013; Hoffman et al., 2018). Recent reports suggest that carnosine has the potential to alleviate pathological conditions associated with ischemia, atherosclerosis and wound healing as well. For instance it has been shown that the treatment with carnosine protects the rodent hearts from ischemia-reperfusion injury (Lee et al., 1999; Baba et al., 2013), and oral supplementation of carnosine (1 g/L) in water to apolipoprotein E-null (*apo*E^-/-^) mice inhibits atherogenesis (Menini et al., 2012, 2015; Barski et al., 2013). Similarly, supplementation of carnosine (1 g/L) improves wound healing responses in rats and *db/db* mice subjected to abdominal and skin wounds (Nagai et al., 1986; Roberts et al., 1998; Ansurudeen et al., 2012). Although the highest concentration of total carnosine in the body is in the skeletal muscle, its contribution to skeletal muscle ischemia has not been examined. To address this knowledge gap, we tested whether the oral carnosine supplementation (1 g/L) post HLI surgery would alter the time course and outcome of ischemic injury in a HLI mice model. This model has been extensively used to study angiogenic responses within the ischemic limb (Niiyama et al., 2009; Zhang M.J. et al., 2016). We found that the carnosine intervention accelerated blood flow recovery, tissue revascularization and muscle regeneration. Our observations that the increase in blood flow and revascularization in carnosine-fed mice is accompanied with increases in both the ambulatory movement and metabolic activity suggests that the increase in angiogenesis is accompanied with the enhanced functionality of the ischemic limb. In addition to our results showing that carnosine intervention improves post ischemic revascularization, our results showing that carnosine pre-treatement enhances blood flow suggests that the prophylactic use of carnosine could accelerate blood flow recovery. This property of accelerating blood flow may be useful in preventing and reducing the severity of acute ischemic events, particularly in diabetic patients who are at a risk of developing PAD. Collectively, these results provide the first line of evidence showing that carnosine promotes post ischemic angiogenic response and improves muscle recovery after ischemic injury.

Local delivery of therapeutics to the site of injury is an important challenge, due to the hypo-perfused nature of the site surrounding the ischemic area. Although systemic delivery is simpler, it could be expected that the therapeutic molecules would be poorly delivered to the site of injury. Outcome of clinical trials in PAD patients injected with HIF-1α viral vectors in the ischemic leg were largely negative or inconclusive, which was largely attributed to the inability of viral vectors to diffuse across the ischemic leg (Creager et al., 2011). Previous studies had demonstrated that the supplementation of β-alanine in humans increases the carnosine levels within the skeletal muscle (Harris et al., 2006; Hoetker et al., 2018), however, the data showing the effects of carnosine feeding on histidyl dipeptide pool within the muscle has not been examined. In this study, we demonstrate that carnosine levels are increased in the ischemic limb of the HLI mice fed carnosine for 3 days, which was accompanied by an increase in the expression of carnosine transporters, PHT1 and PEPT2, and normalization of *TauT* mRNA levels in the ischemic limb after carnosine feeding. Homeostatic levels of carnosine in the skeletal muscle are maintained by a complex interplay between carnosine/histidine transporters (PEPT1, PEPT2, PHT1 and PHT2), the β-alanine transporters (TauT, PAT1, and ATB,^0+^), carnosine synthesis (ATPGD1) and hydrolysis by CNDP1 and 2 (Teufel et al., 2003; Drozak et al., 2010; Everaert et al., 2013a). Therefore, our results showing that the expression of carnosine transporters is enhanced or normalized in the ischemic limb suggests that it might be a positive feedback pathway for the ischemic skeletal muscle, to enhance the carnosine transport within a small span of feeding and prevent tissue damage under ischemic conditions. Our results showed that the mRNA level of Atpgd1, which synthesizes carnosine, was decreased in the ischemic limb after 3 days of HLI. We expected that there would be a concomitant change in carnosine levels; however, carnosine levels remained unchanged after 3 days of HLI. It has been reported that it takes at-least 6–15 weeks for carnosine wash out from the skeletal muscle (Baguet et al., 2009), and ATPGD1 is a sluggish enzyme, suggesting that 3-days of ischemia might not be sufficient to diminish the carnosine levels in the ischemic limb. However, it will be interesting to determine whether prolonged ischemia might decrease its levels and synergize with the ATPGD1 expression. Nevertheless, these finding suggest that carnosine is readily transported across the ischemic tissue, possibly by increase or normalization of the carnosine transporters and thus stimulates post ischemic revascularization. Taken together these observations suggest that the carnosine has the potential to act as a local therapeutic agent that can readily diffuse through the ischemic limb to attenuate ischemic injury.

Angiogenesis is a complex process. It involves multiple interactions between angiogenic and anti-angiogenic factors, endothelial and smooth muscle cells and extracellular matrix (Carmeliet, 2003). HIF-1α signaling pathway has emerged as an attractive target to enhance the post ischemic angiogenic response, which enhances vascular responses, including the production and mobilization of angiogenic factors such as VEGF and IL8 in response to ischemia (Vincent et al., 2000; Warnecke et al., 2003; Semenza, 2003; Milkiewicz et al., 2004). Previous studies found that the adenoviral delivery of HIF-1α to the ischemic limb of aged and diabetic mice increases recovery from ischemic injury and simulates limb perfusion accompanied with greater mobilization of angiogenic cells into circulation and higher VEGF expression (Bosch-Marce et al., 2007; Rey et al., 2009; Sarkar et al., 2009). In our study, animals treated with carnosine and subjected to HLI showed that the HIF-1α was significantly increased in the ischemic limb compared with the untreated contralateral. Furthermore, the VEGF expression was also increased in the ischemic limb of the carnosine treated compared with the untreated HLI mice. That carnosine exerts its beneficial effects due to its ability to enhance HIF-1α and VEGF levels are further supported by our *in vitro* results showing that in hypoxic mouse myoblasts, ODC pretreatment upregulated HIF-1α protein expression and enhanced VEGF level both at the mRNA level and secreted protein. Several studies have shown that the circulating Flk-1^+^/Sca-1^+^ cells are mobilized from the bone marrow in response to ischemia and contribute to revascularization in ischemic tissue (Asahara et al., 1997; Sata, 2006). Although the mobilization of these cells is a complex process, it has been suggested that the proangiogenic cytokine VEGF contributes to bone marrow cell mobilization in the peripheral blood (Asahara et al., 1997). Our results showing that the carnosine treatment upregulated HIF-1α and VEGF levels in the ischemic tissue and stimulated the mobilization of Flk-1^+^/Sca-1^+^ cells into circulation suggest that VEGF might be a downstream signaling molecule that mobilizes these cells into circulation. Collectively, these data support the notion that carnosine has the potential to act as a regulator of angiogenesis, which enhances post ischemic angiogenic response potentially by stabilizing HIF-1α and thereby increasing VEGF secretion and stimulating mobilization of proangiogenic cells to circulation.

**SCHEME 1:**
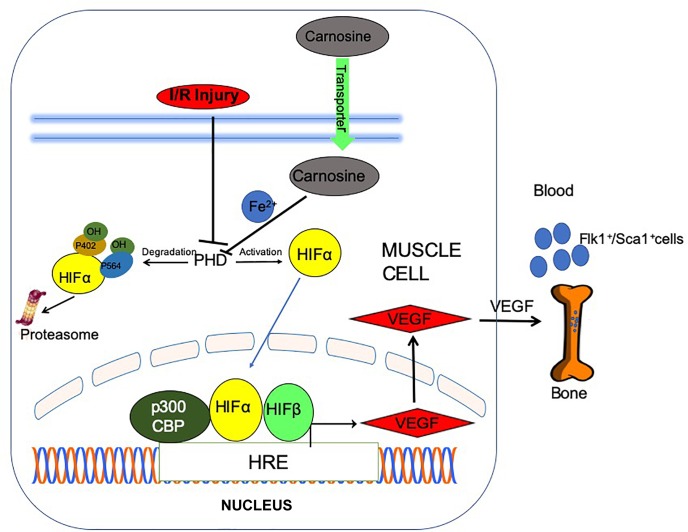
Post ischemic angiogenic signaling in the ischemic muscle is potentiated by carnosine feeding. Bioavailability of carnosine in the ischemic limb is facilitated by increased expression of carnosine transporters (human peptide transporter; PEPT2, carnosine/histidine transporter; PHT1) and normalization of taurine (TauT) transporters. Chelation of (iron) Fe^2+^ by carnosine could inactivate prolyl hydroxylase domain proteins (PHDs), stabilize and translocate hypoxia inducible factor-1 alpha (HIF-1α) to the nucleus, stimulate vascular endothelial growth factor (VEGF) release and mobilize Flk-1^+^/Sca-1^+^ cells into circulation.

Recent reports from our laboratory and others had shown that carnosine has the ability to chelate iron (Fe^2+^) (Canabady-Rochelle et al., 2015; Zhao et al., 2018), inhibit Fe^2+^catalyzed oxidation of lipid phosphatidylcholine liposomes (Baran, 2000), and protects endothelial cells from Fe^2+^-induced cytotoxicity (Zhang S. et al., 2016). Complexes of carnosine and metals are formed through the amino group, peptide nitrogen atom, carboxyl oxygen atom of one peptide molecule and N^π^ atom of the imidazole ring of second peptide molecule (Baran, 2000; Torreggiani et al., 2000a,b, 2003; Mineo et al., 2002). These observations are consistent with our current findings showing that carnosine presents higher Fe^2+^ chelating capacity compared with methylcarcinine in which the N^π^ atom of imidazole ring is methylated and lacks the carboxylate group essential for metal chelation (Torreggiani et al., 2000b). Numerous studies had shown that prolyl hydroxylase domain proteins (PHDs) proteins play a critical role in regulation of HIF-1α. PHDs belong to a family of iron and 2-oxoglutarate-dependent dioxygenases, which requires molecular oxygen, 2 oxoglutarate and iron for catalytic activity. Because PHDs requires iron to catalyze HIF-1α prolyl hydroxylation, compounds that compete for endogenous Fe^2+^ such as deferoxamine and cobalt chloride are widely used to inhibit PHD activity and prevents HIF-1α proteasomal degradation (Wang et al., 1995; Bruick and McKnight, 2001; Ivan et al., 2001; Jaakkola et al., 2001; Milkiewicz et al., 2004; Ikeda et al., 2011). The physiological significance of carnosine as Fe^2+^ chelator is supported by our data showing that enhancing carnosine levels in C2C12 cells by carnosine or ODC treatment upregulated HIF-1α protein expression and VEGF release in the media, whereas pretreatment with the dipeptide analog methylcarcinine, which lacks the ability to chelate Fe^2+^, failed to upregulate HIF-1α levels or VEGF release, under normoxic nor hypoxic conditions. Hence, it is to be anticipated that the chelation of Fe^2+^ by carnosine may inactivate PHDs in the ischemic limb, and thus could be one way of promoting angiogenesis in the ischemic limb. However, further investigation is needed to determine whether carnosine could directly enhance the HIF-1α protein expression. Given that carnosine is a multifunctional dipeptide that sequesters lipid peroxidation products (Aldini et al., 2002), our data do not exclude the possibility that carnosine may provide muscle protection by other mechanisms. Recent reports show that HNE protein adducts are accumulated in the skeletal muscle of PAD patients and ischemic limb of mice subjected to HLI (Pipinos et al., 2006, 2008). These aldehydes impair a variety of angiogenic processes, such as decrease in VEGF expression (Kumagai et al., 2002; Kaneko et al., 2011) and mobilization of Flk-1^+^/Sca-1^+^ double positive cells into the peripheral blood (Wheat et al., 2011). Conversely activation of pathways that detoxify HNE preserves the proangiogenic potential in human endothelial cells (Solito et al., 2013). Similarly, in PHD3 deficient mice, ischemic injury induced by HLI resulted in reduced formation of aldehyde protein adducts (Aragones et al., 2008). Nevertheless, taken together, our results suggest that carnosine could be a unique endogenous non-toxic molecule, which readily diffuses across the ischemic skeletal muscle augments HIF-1α/VEGF signaling axis potentially by chelating Fe^2+^ and thus could enhance angiogenic responses in the ischemic skeletal muscle (Scheme 1).

In summary, we in this study provide evidence for a new application of carnosine functionality in the setting of HLI, wherein carnosine supplementation improves post ischemic revascularization. Because carnosine is a natural dipeptide and a food constituent, it is safe for consumption and supplementation in humans, and thus it is readily available for clinical testing as a potential therapeutic intervention in PAD/CLI patients. Significantly, our results also highlight the important physiological role of carnosine in ameliorating tissue injury and promoting revascularization and thereby add a new facet to the biology of histidyl dipeptides, which are widely distributed in nature.

## Ethics Statement

All treatments and protocols were approved by the University of Louisville Institutional Animal Care and Use Committee.

## Author Contributions

AAB, DZ, LG, YZ, DH, HZ, JZ, DP, and VK performed the experiments and analyzed the data. SB and DC performed the experiments, analyzed the data, and conceptualized the study design. AB, MW, and CN helped with study design and concept. AK performed the experiments.

## Conflict of Interest Statement

The authors declare that the research was conducted in the absence of any commercial or financial relationships that could be construed as a potential conflict of interest.

## Abbreviations

ATPGD1: carnosine synthase
CLI: critical limb ischemia
CNDP2: carnosinase
HIF-1α: hypoxia inducible factor-1alpha
HLI: hind limb ischemia
ODC: octyl D carnosine
PEPT2: peptide transporter
PHDs: prolyl hydroxylase domain proteins
PHT1: peptide/histidine transporter
TauT: taurine transporter
VEGF: vascular endothelial growth factor
