# Supplementary material for: Carnosine Supplementation Enhances Post Ischemic Hind Limb Revascularization
Source: Front Physiol. 2019 Jul 2;10:751. doi: 10.3389/fphys.2019.00751 (PMC6614208; doi:10.3389/fphys.2019.00751)
Supplement: Supplementary file 1 [file Table_1.DOCX]

**Supplemental Methods**

**Synthesis of methyl carcinine**

*Synthesis of 7,8-dihydroimidazo[1,5-c](45) pyrimidin-5(6H)-one (2)*

In a round bottom flask Histamine dihydrochloride (**1**, 3.0 g, 16.3 mmol), 1,1'- Carbonyldiimidazole (2.8 g, 17.1 mmol) were added in DMF (50 mL) at room temperature. Reaction mixture was heated at 60^o^C for 6 h to evaporate DMF. The crude material was purified with silica gel column chromatography to get the desired compound (1.8 g, 80.7%) as a white solid compound. ^1^H NMR (CDCl_3_, 400 MHz): *δ* 8.15 (s, 1H), 6.86 (s, 1H), 5.89 (bs, 1H), 3.57–3.52 (m, 2H), 3.02–2.95 (m, 2H) ppm.

1. *Synthesis of 2-methyl-5-oxo-5,6,7,8-tetrahydroimidazo[1,5-c] pyrimidin-2-ium iodide (3):*

In a round bottom flask ,7,8-dihydroimidazo[1,5-*c*] pyrimidin-5(6*H*)-one (**2**, 2.0 g, 14.6 mmol) and methyl iodide (3.6 mL, 58.3 mmol) were added in acetonitrile (50 mL) at room temperature. The reaction mixture was heated at 80 ^o^C for 16 h, and evaporated to dryness to get the desired compound (3.9 g, 96%) as white solid compound. ^1^H NMR (D_2_O, 400 MHz): *δ* 9.16 (s, 1H), 7.26 (s, 1H), 3.86 (s, 3H), 3.56–3.52 (m 2H), 3.04–3.00 (m, 2H) ppm.

*(c) Synthesis of 2-(1-methyl-1H-imidazol-4-yl)ethanamine hydrochloride (4):*

In a round bottom flask 2-methyl-5-oxo-5,6,7,8-tetrahydroimidazo[1,5-c]pyrimidin-2-ium iodide (**3**, 2.0 g, 7.2 mmol) and 4N HCl (20 mL) was refluxed for 4 h, evaporated to dryness, and the solid extract was washed with hexanes and dried to get the desired compound, 2-(1-methyl-1*H*-imidazol-4-yl) ethanamine hydrochloride, (**4**, 0.90 mg, 78%) as yellow solid compound. ^1^H NMR (D_2_O, 400 MHz): *δ* 8.55 (s, 1H), 7.28 (s, 1H), 3.79 (s, 3H), 3.26–3.22 (m, 2H), 3.06–3.02 (m, 2H) ppm.

*(d) Synthesis of tert-butyl 3-(2-(1-methyl-1H-imidazol-4-yl) ethylamino)-3-oxopropylcarbamate (6):*

To a solution of 2-(1-methyl-1*H*-imidazol-4- yl) ethanamine hydrochloride (**4**, 2.5 g, 15.5 mmol) in dry DCM (60 mL) under nitrogen, was added Boc-b-alanine (**5**, 3.2 g, 17.0 mmol), EDC (N-(3-Dimethylaminopropyl)-N′-ethylcarbodiimide) (3.26 g, 17.02 mmol) and N-methylmorpholine (6.8 mL, 61.8 mmol via syringe). The contents were stirred at room temperature for 48 h, and slowly diluted into iced water and extracted with DCM. The organic phase was dried and evaporated under vacuum. Purification was done by silica gel column chromatography with 5% MeOH in DCM to get tert-butyl 3-(2-(1*H*-imidazol-5-yl) ethylamino)-3-oxopropylcarbamate (**6**, 2.2 g, 48%) as a white solid compound.^1^H NMR (D_2_O, 400 MHz): *δ* 7.43 (s, 1H), 6.79 (s, 1H), 3.55 (s, 3H), 3.33–3.29 (m, 2H), 3.21–3.17 (m, 2H), 2.63–2.57 (m, 2H), 2.27–2.23 (m, 2H), 1.30 (s, 9H) ppm.

*(e) Synthesis of 3-amino-N-(2-(1-methyl-1H-imidazol-4-yl) ethyl) propanamide hydrochloride (7):*

Tert-butyl 3-(2-(1-methyl-1H-imidazol-4- yl) ethylamino)-3-oxopropylcarbamate (**6**, 2.1 g, 7.09 mmol) was stirred in 4N HCl in dioxane (30 mL) for 2 h at room temperature evaporated to dryness and the obtained solid was washed with hexanes and dried again to get 3-amino-*N*-(2-(1- methyl-1*H*-imidazol-4-yl)ethyl) propanamide dihydrochloride (**7**, 1.4 g, 85%) as white solid compound. ^1^H NMR (D_2_O, 400 MHz): *δ* 8.46 (s, 1H), 7.15 (s, 1H), 3.76 (bs, 3H), 3.41–3.37 (m, 2H), 3.15–3.11 (m, 2H), 2.82–2.79 (m, 2H), 2.57–2.53 (m, 2H) ppm. ^13^C NMR (D_2_O, 100 MHz): *δ* 172.2 (CO), 134.6 (CH), 131.4 (CH), 120.3 (C), 38.0 (CH_2_), 35.7 (CH_2_), 35.5 (CH_2_), 31.9 (CH_2_), 24.1 (CH_3_) ppm.
